# MicroRNA Modulation by Dietary Supplements in Obesity

**DOI:** 10.3390/biomedicines8120545

**Published:** 2020-11-27

**Authors:** Tiziana Filardi, Claudia Sabato, Carla Lubrano, Carmela Santangelo, Susanna Morano, Andrea Lenzi, Silvia Migliaccio, Elisabetta Ferretti, Giuseppina Catanzaro

**Affiliations:** 1 Department of Experimental Medicine, Faculty of Medicine and Dentistry, “Sapienza” University of Rome, Viale Regina Elena 324, 00161 Rome, Italy; tiziana.filardi@uniroma1.it (T.F.); carla.lubrano@uniroma1.it (C.L.); susanna.morano@uniroma1.it (S.M.); andrea.lenzi@uniroma1.it (A.L.); elisabetta.ferretti@uniroma1.it (E.F.); giuseppina.catanzaro@uniroma1.it (G.C.); 2 Gender Specific Prevention and Health Unit, Center for Gender-Specific Medicine, Istituto Superiore di Sanità, Viale Regina Elena 299, 00161 Rome, Italy; carmela.santangelo@iss.it; 3 Section of Health Science, Department of Movement, Human and Health Sciences, University of Rome “Foro Italico”, 00135 Rome, Italy; silvia.migliaccio@uniroma4.it

**Keywords:** obesity, dietary supplements, diet, microRNA, polyphenols, fatty acids, weight loss, obesity treatment, adipose tissue

## Abstract

The prevalence of obesity has dramatically increased over the last decades. Weight loss obtained through diet and exercise leads to a significant decrease in morbidity and mortality. Recently, there has been growing interest in the possible beneficial effects of dietary supplements (DSs), including polyphenols, fatty acids, and other plant-derived substances, as adjuvants in the management of obesity and metabolic diseases. Specifically, polyphenols, widely spread in vegetables and fruits, significantly modulate adipose tissue activities, contrasting inflammation and improving insulin sensitivity in preclinical and clinical studies. Remarkably, polyphenols are involved in complex microRNA networks, which play crucial roles in metabolic processes. The administration of different polyphenols and other plant-derived compounds led to significant changes in the microRNA expression profile in peripheral tissues in a growing number of preclinical studies. In particular, these compounds were able to revert obesity-induced microRNA dysregulation, leading to the inhibition of adipogenesis and the induction of weight loss. Furthermore, through microRNA modulation, they attenuated key metabolic alterations, including insulin resistance and lipid anomalies, in animal models of obesity. Some of them were also able to reduce proinflammatory cytokines in adipose tissue. The aim of this review is to summarize current evidence about the effect of plant-derived DSs on microRNA expression in obesity.

## 1. Introduction

Over the last decades, the prevalence of obesity has consistently grown, becoming a pandemic health concern. It is estimated that obesity and overweight affect up to 50% of the adult population worldwide [1]. Excess body weight is one of the most important risk factors for all-cause morbidity and mortality [2]. In particular, in line with the dramatic spread of these conditions, the occurrence of non-communicable diseases, such as type 2 diabetes (T2D), cardio- and cerebro-vascular diseases (CVD), respiratory diseases, and cancer has consistently risen [3,4,5], accounting for over 80% of all premature deaths [1]. 

The main treatment strategies to achieve weight loss include lifestyle changes, consisting in promoting healthy dietary patterns to reduce energy intake and enhancing physical activity to increase energy expenditure [6]. Besides lifestyle modifications, several pharmacologic agents are currently available for obesity management, even though conflicting results in terms of efficacy, durability of weight control, and adverse effects have been reported in clinical trials [7]. In spite of the overall improvement in the therapeutic strategies for weight loss, the management of obesity is still challenging. In the last years, the potential role of natural phytochemicals for weight management has gained growing attention [8] and the use of dietary supplements (DSs) as adjuvant treatment for obesity and metabolic diseases has greatly increased [9]. DSs are defined as “products that supplement diet, with or without additional nutritional value, which contain one or more of the following ingredients: a vitamin, a mineral, a herb or other botanical, an amino acid, a dietary substance to supplement diet by increasing the total dietary intake, or a concentrate, metabolite, constituent, extract, or combination of any reported ingredient” [10,11]. DSs have become an attractive therapeutic option due to their generally low toxicity profile and easy access to the general population. 

Over the last decades, there has been growing interest in microRNA, a class of small non-coding RNAs that modulate gene expression, as regulators of metabolic processes and biomarkers of disease. In particular, a wide number of studies have depicted a dysregulation of microRNA expression in obesity and metabolic diseases [12,13,14]. A wide number of dysregulated microRNA target genes involved in pathways critically associated in glucose and lipid metabolism, energy homeostasis, inflammation, immunity, and endothelial function have also emerged, helping unravel the pathophysiological mechanisms underlying obesity and obesity-linked metabolic diseases [13]. Furthermore, circulating and tissue microRNA expression have been found to be significantly modified by different weight loss interventions, including different dietary patterns, physical activity programs, and bariatric surgery [13], suggesting that weight loss and the related metabolic changes might be mirrored by significant modifications in the microRNA signature. Similarly, a wide variety of DSs, such as polyphenols and other plant compounds, have been shown to regulate microRNA expression in adipose tissue in preclinical models of diet-induced obesity. Therefore, microRNA might be critically involved in the outcome of weight loss interventions. In light of this, a novel approach to obesity treatment is focused on customized nutritional interventions, which take into account not only the phenotype but also genetic and epigenetic data, helping personalize the management of this complex condition [15]. 

The aim of this review is to summarize the current evidence on the effects of DSs with potential or demonstrated anti-obesity properties on the circulating and tissue microRNA expression profile, to help understand the complex pathophysiological mechanisms underlying obesity and to possibly identify novel candidate biomarkers of metabolic modifications and co-morbidities, such as T2D and hyperlipidemia.

## 2. General Aspects of MicroRNAs 

MicroRNAs are a class of small (19-25 nucleotides), endogenous, and non-coding RNAs that regulate eukaryotic gene expression [16]. They control gene expression via Watson–Crick base-pairing to the 3′ untranslated regions (3′UTRs) of target messenger RNAs (mRNAs) by inhibiting translation and by affecting mRNA stability and degradation [16,17,18]. A single microRNA may target more than 100 mRNAs as well as multiple microRNAs targeting the same gene [19,20].

MicroRNA genes are transcribed by RNA polymerase II (RNA pol II) from intronic regions of non-coding or coding transcripts and exonic regions as longer primary transcripts (pri-miRNAs) and double stranded RNAs (dsRNAs), containing a local stem-loop structure. The pri-miRNA is sequentially cleaved into shorter intermediates by ribonuclease III enzymes: Drosha, Dicer, and dsRNAs-specific nucleases [21,22].

In the nucleus, a heterotrimeric complex termed microprocessor, containing one molecule of the Drosha endonuclease and two molecules of its partner protein Di George syndrome critical region (DGCR8), crops the pri-miRNAs into a shorter hairpin-structured precursor (pre-miRNA) of 65–70 nucleotides in length, bearing 2 nucleotides 3′ overhang, characteristic of RNase III-mediated cleavage. The nuclear transport receptor exportin 5 recognizes the pre-miRNA overhang and mediates the nuclear export of pre-miRNA to the cytoplasm in a Ran-GTP-dependent process, where the pre-miRNA is further processed into a mature miRNA duplex of 22 nucleotides by the RNase III-type endonuclease Dicer in association with TAR RNA-binding protein (TRBP) [23,24]. Following Dicer processing, the miRNA duplex is released and subsequently loaded into argonaute (Ago) family proteins (Ago 1–4 in human) along with chaperone proteins (HSC70/HSP90) to form an effector complex called RNA-induced silencing complex (RISC) in an ATP-dependent manner. The process culminates in the stable association of only one of the two strands with the Ago effector proteins (Ago2). On the basis of the relative thermodynamic stability of the two ends of the microRNA duplex, the less stable strand terminus at the 5ʹ side will be typically selected as the guide strand, whereas the other strand (passenger strand*) will be degraded [16,21,23]. The mature miRISC recognizes and regulates mRNA target binding by base pairing, with the degree of microRNA-mRNA complementarity being the determinant regulatory mechanism. The perfect complementarity triggers the endonucleolytic mRNA cleavage and the subsequent translation repression, whereas the imperfect one matches mRNA deadenylation and decapping [25]. 

MicroRNAs exert crucial roles in almost every cellular process by modulating the biological physiologic homeostasis; however, changes in their expression have been observed in human pathologies. MicroRNAs may not only act within cells but also in the extracellular space. Indeed, they have been found in extracellular body fluids, such as serum, plasma, saliva, breast milk, and urine [26].

MicroRNAs are released from cells in membrane-bound vesicles, such as exosomes, microvesicles, and apoptotic bodies, which protect them from blood RNases activity and increase their stability, as well as being associated in complexes with Ago2 proteins or high-density lipoproteins (HDLs) [27,28,29,30].

They act as hormone-like molecules and exert important roles in cell-to-cell communication; these findings have suggested their use as informative biomarkers of physio-pathological status [31]. 

The pivotal role of microRNAs in metabolic homeostasis and their implication in metabolic diseases (e.g., metabolic syndrome, T2D) has been suggested in several studies.

MiR-122, an abundant liver-specific microRNA, was shown to affect hepatic cholesterol and lipid metabolism, as its inhibition in normal and high-fed mice was associated with a significant reduction in hepatic steatosis and plasma cholesterol levels [32,33]. MiR-33a/b were reported to control cholesterol/lipid homeostasis together with their host gene products, the sterol regulatory element-binding protein (SREBP), through the regulation of the ATP-binding cassette A1 (ABCA1) cholesterol transporter and fatty acid (FA) β-oxidation genes, such as carnitine O-octanoyltransferase (CROT), carnitine palmitoyltransferase 1A (CPT1A), and hydroxyacyl-CoA dehydrogenase–3-ketoacyl-CoA thiolase–enoyl-CoA hydratase β-subunit (HADHB) [34,35]. Furthermore, the loss of miR-33 leads to the development of obesity and insulin resistance in target tissues, including the liver, white adipose tissue (WAT), and skeletal muscle, in an miR-33 deficient mouse fed a high-fat diet (HFD) [36]. Several microRNAs have been identified to regulate the responses to insulin and glucose homeostasis in the liver, muscle, and adipose tissue. Increased miR-103 and miR-107 expression levels impaired glucose homeostasis and insulin sensitivity in the livers of leptin-deficient (ob/ob) and diet-induced obese (DIO) mice [37]. Similarly, overexpression of miR-143 [38], let-7 [39], and miR-29b [40] has been reported to inhibit insulin signaling and to impair glucose tolerance.

Moreover, numerous microRNAs have been demonstrated to regulate adipocyte differentiation and function [41,42].

## 3. Effects of Dietary Supplements on MicroRNA Expression in Obesity

Recently, multiple DSs, including polyphenols (Table 1), fatty acids (FAs), and other plant-derived compounds (Table 2), have been shown to regulate microRNA expression in peripheral tissues in animal models of diet-induced obesity. Although clinical evidence regarding the effect of these supplements on microRNA expression is still lacking, data from preclinical studies helped in identifying novel potential biomarkers of metabolic alterations in obesity.

### 3.1. Polyphenols 

Polyphenols are a heterogeneous group of plant-derived substances, widely spread in vegetables and fruits as well as in other products, such as tea, coffee, chocolate, cereals, wine, olives, oils, and spices [55]. The chemical structure of polyphenols includes a variable number of phenolic rings that bind to other structural elements. The major components of this class are flavonoids, including anthocyanins, proanthocyanidins, flavanones, flavones, flavonols, and isoflavonoids, and non-flavonoids, such as resveratrol [55,56]. Flavonoids are the most abundant polyphenols in the Mediterranean diet [57]. A growing body of evidence has suggested a conceivable link between polyphenol intake and multiple beneficial health effects. In particular, the role of dietary polyphenol in the prevention of non-communicable diseases has been widely studied in the last years. Interestingly, polyphenols are able to interact with different cellular components and molecules, including microRNAs, and are therefore known to exert multiple functions, being involved in several molecular pathways [58]. Remarkably, polyphenols seem to play a role in glucose homeostasis regulation, being able to contrast intestinal glucose absorption, to promote glucose uptake in peripheral tissues, to improve pancreatic β-cell function, and to modulate hepatic glucose release [59]. Specifically, polyphenols exerted anti-diabetic effects by enhancing β-cell function and improving glucose tolerance in vitro, in both animal and human studies [60,61,62,63,64,65,66]. Furthermore, they have been reported to reduce inflammation [67,68] and to modulate adipocyte function. In particular, resveratrol exposure of 3T3-L1 adipocytes induced adiponectin expression, reduced leptin levels [69,70], and inhibited the expression of proinflammatory cytokines (Interleukin-6and Tumor necrosis factor-α) [71]. Similarly, the flavonoid fisetin increased adiponectin gene transcription in mouse adipocytes, by inducing PPAR activation and Sirtuin-1-deacetylase activity [72,73]. Altogether, these data suggest that polyphenols significantly modulate adipose tissue function, reducing inflammation and improving insulin sensitivity, therefore being potential valuable candidates as therapeutic agents in obesity and metabolic diseases. However, it should be highlighted that evaluating the effects of polyphenols in this field is rather challenging, due to their complex interplay with the human body. Indeed, the level of human exposure to polyphenols is influenced by food-related factors, such as polyphenol bioavailability and content in food, and other factors, such as genetics and gut microbiota activity [74]. It is well established that polyphenols are widely modified and bio-transformed once introduced into the gastrointestinal tract, generating different active metabolites [75,76] whose plasma concentrations are generally low (nmol/L) [76]. Moreover, some flavonoids are substantially modified by microbiota in the large intestine [77]. In light of this, it is worth noting that many studies might have tested the wrong compound at the wrong concentration, leading to misleading and inconsistent results and conclusions. Extraction and purification of polyphenols for nutraceutical use is another key issue that might have consistently influenced the results of preclinical studies and might limit polyphenol use in clinical practice. In fact, the overall effect of polyphenol-rich preparations might have been modulated by the contextual presence of other compounds with synergistic action [78].

Polyphenols have been shown to be involved in complex microRNA networks. In particular, an increasing number of preclinical studies have reported changes in the microRNA expression in adipose tissue after exposure to different polyphenols, mirroring significant metabolic changes (Table 1). 

Specifically, Qin et al. observed that supplementation with the synthetic flavonoid 3-O-[(E)-4-(4-cyanophenyl)-2-oxobut-3-en-1-yl] kaempferol (Fla-CN) led to a dose- and time-dependent reduction in weight gain and visceral fat mass in high-fat diet (HFD)-fed mice, compared with non-treated HFD-fed controls, independently of food intake. In addition, HFD-induced lipid alterations and insulin resistance were significantly attenuated in the Fla-CN treated group. Accordingly, a reduction in leptin and proinflammatory cytokines was observed after treatment, while adiponectin levels significantly rose. Interestingly, miR-27 expression in the liver and in adipose tissue increased in the Fla-CN group, compared to the non-treated group. The expression of four miR-27 target genes involved in adipogenesis, namely peroxisome proliferator activated receptor-γ (PPAR-γ), CCAAT-enhancer-binding protein α (C/EBP α), sterol regulatory element-binding protein-1c (SREBP-1c), and fatty acid synthase (FAS), was downregulated in adipose tissue in Fla-CN-treated HFD mice, suggesting that Fla-CN might inhibit adipogenesis through miR-27 upregulation, promoting anti-obesity and anti-diabetic effects. Remarkably, a comparable effect on miR-27 expression was obtained with metformin administration in the same model [43]. 

Jeon et al. explored the effects of fisetin [44], a natural flavonol with confirmed antioxidative and anti-inflammatory properties [79]. In detail, the authors maintained four-week-old male C57BL/6J mice on three different dietary plans: normal diet from the American Institute of Nutrition AIN-76A, HFD, and HFD supplemented with 0.05% fisetin (HFD+F) for 10 weeks and evaluated the expression pattern of selected microRNAs in mice livers at the end of the study. The expression level of five microRNAs (miR-22*, miR-146a, miR-146b, miR-802, and miR-378) was significantly increased in HFD compared to ND-fed mice, and fisetin supplementation was able to completely reverse these results. Furthermore, the authors demonstrated that fisetin was able to downregulate both miR-378 and its host-gene proliferator-activated receptor-γ coactivator-1 β (PGC-1β) in the mouse liver, and that the putative effect of miR-378 on the modulation of lipid metabolism genes could be due to its ability to repress the nuclear respiratory factor 1 (NRF-1), a transcription factor with a pivotal role in the development of fatty liver [44,80]. Another study focused on the effect of long-term consumption of a grape seed proanthocyanidin extract (GPSE) on the liver expression levels of miR-33a and miR-122, previously described as major regulators of lipid metabolism in this tissue [81]. To demonstrate that dietary proanthocyanidins were involved in miR-33a and miR-122 modulation, Baselga-Escudero et al. fed 36-week-old female Wistar rats with a standard (STD) or a HFD for 15 weeks. At the end of this first period, the HFD-fed rats were divided into four groups and maintained on HFD with different doses of GSPE supplementation (from 5 to 50 mg/kg) for another three weeks. The authors demonstrated that, at all the administered doses, GSPE significantly reduced miR-33a and miR-122. Moreover, high doses of GSPE reduced plasma triglycerides and total cholesterol, although not affecting body weight, suggesting that dietary proanthocyanidin-rich foods may be beneficial in counteracting both obesity and obesity-associated risk factors [45]. 

Gracia et al. evaluated changes in microRNA expression in visceral adipose tissue (VAT) after administration of resveratrol in a rat model of diet-induced obesity. In resveratrol-treated rats, body weight, as well as VAT and subcutaneous adipose tissue (SAT) masses, were reduced, compared to the control group. The expression of miR-211-3p, miR-1224, and miR-539-5p significantly increased, while miR-511-3p decreased after treatment. In bioinformatics analysis, the predicted target genes of the differentially expressed microRNAs were PPAR-γ, hormone-sensitive lipase (HSL), and SP1 transcription factor (SP1), which are critically involved in FA metabolism in adipose tissue [46]. Specifically, the SP1 gene is a confirmed target gene of miR-539-5p, and miR-1224 has been previously shown to be involved in SP1 regulation [82]. Notably, the synergistic action of SP1 and SREBP-1 induces the expression of FAS, which promotes de novo lipogenesis [83]. In resveratrol-treated rats, protein expression of SP1 and SREBP-1 was significantly reduced, suggesting that resveratrol might inhibit adipogenesis through the upregulation of miR-539-5p and the suppression of SP1 and SREBP-1 [46]. 

Tian et al. investigated the anti-obesity properties of the polyphenol compound curcumin [47]. This plant-derived substance has been widely studied in different pathological contexts, due to its antidiabetic, anti-inflammatory, antioxidant, neuroprotective, hepatoprotective, antiangiogenic, immunomodulatory, and anti-hypertensive effects, and has recently emerged as a therapeutic agent for several conditions [84]. In HFD-fed mice, miR-17-5p expression was enhanced in epididymal adipose tissue and its overexpression showed obesogenic effects. Specifically, miR-17-5p upregulation promoted 3T3-L1 adipogenic differentiation, which was instead reduced by miR-17-5p inhibition. Similarly, curcumin was able to attenuate 3T3-L1 cells’ adipogenic differentiation through the suppression of miR-17-5p [47]. 

Otton et al. reported that green tea (GT) derives from *Camellia sinensis*, a plant that contains high concentrations of flavonoids, such as catechins, and other polyphenolic compounds, and evaluated the beneficial effects of GT on the microRNA expression profile of mice epididymal WAT (eWAT). Specifically, C57BL/6 mice were firstly treated for four weeks with standard diet or HFD, then with water or GT for another 12 weeks. At the end of the study, they were randomly assigned to four diet interventions: mice fed with standard diet and gavage with water (CON) or gavage with GT; and mice fed with high-fat diet and gavage with water (OB) or gavage with GT (OB+GT). Mice fed with HFD increased body weight, proinflammatory cytokines (TNF-α, IFN-γ, IL-6, MCP-1, IL-1β, IL-17), and the adiposity index, whereas GT reversed obesity, restored inflammatory cytokines to basal levels, and reduced insulin resistance. MicroRNA array analysis conducted in mice eWAT revealed microRNA modulation in each dietary intervention (GT, OB, and OB+GT). Seven microRNAs (miR-34a, miR-211, miR-155, miR-132, miR-335, miR-802, miR-455) exhibited a pattern of increased expression in obese mice and decreased expression after the GT treatment. However, only three microRNAs, namely miR-155, miR-221, and miR-335, exhibited the same expression pattern in an independent validation cohort, confirming them as GT-sensitive microRNAs. Moreover, miR-155 and miR-335 were further chosen for in vivo and in vitro studies with microRNA mimics or inhibitors, revealing that miR-355 but not miR-155 correlated positively with changes in adipocyte gene expression in obese mice [48]. More recently, Torres et al. demonstrated that the hepatoprotective role of GT was partly mediated by microRNA modulation. C57BL/6 mice were subjected to the same treatment scheme adopted by Otton et al. At the end of 16 weeks, mice fed with HFD and GT showed a reduced body weight accompanied by reduced adiposity and HOMA index. In addition, when compared with HFD-fed mice, the GT-supplemented mice showed an increase in lipid catabolism genes with a parallel reduction in macrovesicular steatosis, triglycerides, and cholesterol content. The authors investigated miR-34a and miR-194 modulation in the HFD-fed mice and demonstrated that the expression of these microRNAs in the liver was reversed after the addition of GT. In particular, GT induced a downmodulation of HFD-induced miR-34a levels and an increase in miR-194 levels. Moreover, the expression of several target genes involved in lipid-β oxidation, and lipid and cholesterol metabolism (SIRT1, PPAR-α, insulin-induced gene 2 (INSIG2) for miR-34a; apolipoprotein A-5 (APOA5) and 3-hydroxy-3-methylglutaryl-CoA-synthase 2 (HMGCS2) for miR-194), was inversely correlated with miR-34a and miR-194 expression. Cell experiments on the human hepatoma cells (HepG2) confirmed these results and corroborated the idea that, at least for miR-194, the GT effect is due to TNF-α repression [49].

### 3.2. Fatty Acids 

FAs are a fundamental source of energy and regulate important metabolic functions. In the last decades, several FA compounds have emerged as candidate anti-obesity drugs, due to their body fat-lowering properties (Table 2). Among them, the effect of the administration of conjugated linoleic acid (CLA), a FA naturally found in the milk and meat of ruminant animals [85], has been evaluated in HFD-fed mice. Specifically, the expression of miR-27a in adipose tissue was lower in HFD than in normal-fat diet (NFD) mice, while a 4-week treatment with CLA reversed this condition. These findings suggest that HFD might suppress miR-27 expression in adipose tissue, leading to the enhanced activity of several genes that promote adipocyte differentiation and fat accumulation. In light of this, dietary agents that are able to target miR-27, enhancing its expression, might effectively inhibit adipogenesis, promote weight loss, and improve metabolic alterations in obesity [50]. Similarly, Parra et al. evaluated the effect of CLA treatment on microRNA expression in C57BL/6J mice. Two independent experiments with different dietary regimes (standard-fat or HFD) and different doses of CLA were carried out. After CLA treatment, the expression levels of selected microRNAs (miR-143, miR-103, miR-107, miR-221, miR-222), involved in adipocyte differentiation and associated with obesity were measured by RT-qPCR in retroperitoneal adipose tissue (rWAT) of mice fed with a standard-fat diet or with a HFD. Results showed that metabolic status and microRNA expression were modified in response to CLA treatment, together with a 67% reduction in rWAT. Among the assayed microRNAs, only miR-107 showed a significant dose-dependent decreased expression in both dietary arms, while miR-221 expression significantly increased in the HFD group at the highest CLA dose. A significantly increased expression of miR-222 was found at the highest CLA dose of the standard-fat diet, while the same tendency did not reach statistical significance in the HFD group. However, it is worth noting that CLA treatment was able to modulate the expression of the selected microRNAs in adipose tissue, proportionally to the entity of the remodeling of adipose tissue [51].

Chen et al. demonstrated instead the important role of oleanolic acid (OA), a natural compound with anti-obesity and antihyperglycemic effects, in attenuating obesity and in the improvement of glucose metabolism mediated by the G protein-coupled receptor of bile acids TGR5/miR-26a axis. Eight-week-old C57BL/6 wild-type (WT) and TGR5-/- mice were fed with control and HFD for 8 weeks. The last group was further divided into two subgroups fed with HFD only or with HFD plus OA for another 8 weeks. OA treatment induced not only a significant upregulation of miR-26a in the livers of WT mice but also a strong increase of this microRNA in Kupffer cells, the liver-resident macrophages, when compared to vehicle-treated cells (CON). This effect could be reversed in Kupffer cells derived from JNK-/- mice or by the concurrent treatment with a JNK inhibitor. Of note, both TGR5 and miR-26a are critical players in obesity, glucose homeostasis, and lipid metabolism [86,87,88] and the disclosure of their interaction could be exploited to develop new approaches for the treatment of metabolic diseases [52]. 

### 3.3. Other Compounds 

Ahn et al. evaluated the effects of the dietary supplement zerumbone, a compound of wild ginger, on HFD-fed mice. Epididymal adipose tissue weight and adipocyte size were significantly reduced in the zerumbone-supplemented mice compared to controls. Furthermore, zerumbone was able to attenuate HFD-induced dyslipidemia and insulin resistance. Adipogenic differentiation was suppressed by zerumbone in the 3T3-L1 cell line. Interestingly, miR-146b was significantly upregulated in adipose tissue of HFD-fed mice and zerumbone was able to attenuate the overexpression of miR-146b both in adipose tissue and in differentiated 3T3-L1 cells. It has been reported that miR-146b negatively modulates SIRT1 during adipogenesis. Thus, zerumbone acts as a negative regulator of miR-146b, increasing SIRT1 expression and downregulating lipogenesis-related genes. Pharmacological activation of SIRT1 might therefore have beneficial effects in treating obesity and metabolic diseases [53]. 

It has been observed that SIRT1 is activated by an increased intracellular NAD+/NADH ratio in WAT [89]. The plant-derived ß-lapachone (BLC) is a naphthoquinone with anti-obesity properties, due to its ability to induce intracellular oxidation of NADH to NAD+ [90,91]. Choi et al. evaluated the effect of BLC supplementation on body weight and fat accumulation in HFD-fed mice [54]. After 11 weeks, the HFD group supplemented with high-dose BLC had a significantly lower body weight compared to the low-dose BLC group and control HFD group, independently of food intake. In addition, a reduction in white adipose tissue weight and adipocyte size was observed with BLC treatment, together with a decrease in blood glucose, insulin, and leptin. An increase in energy expenditure was also observed in the high-dose BLC HFD group. Interestingly, the administration of BLC exerted fat-browning effects in SAT, leading to an upregulation of brown-specific genes and proteins. Specifically, miR-382 expression was significantly downregulated in SAT of BLC-supplemented mice, while mRNA and protein expression of its predicted target iodothyronine deiodinase 2 (Dio2) increased. It is well established that the activation of Dio2 enhances energy expenditure, activating the conversion of thyroxin (T4) to triiodothyronine (T3) [92]. In functional assay, the activation of miR-382 significantly downregulated Dio2, uncoupling protein 1 (UCP1) and PPAR-γ mRNA and protein, confirming that miR-382 is an inhibitor of fat browning in WAT. On the contrary, treatment with BLC in vitro significantly reversed this condition. The dissipation of energy through heat production is the main function of brown adipose tissue and largely depends on UCP1 mitochondrial expression. Adipocytes that express browning-specific genes, such as UCP1, in response to several stimuli, have been found in human SAT and are novel therapeutic targets in obesity [54].

## 4. Conclusions and Future Perspectives

**Figure 1:**
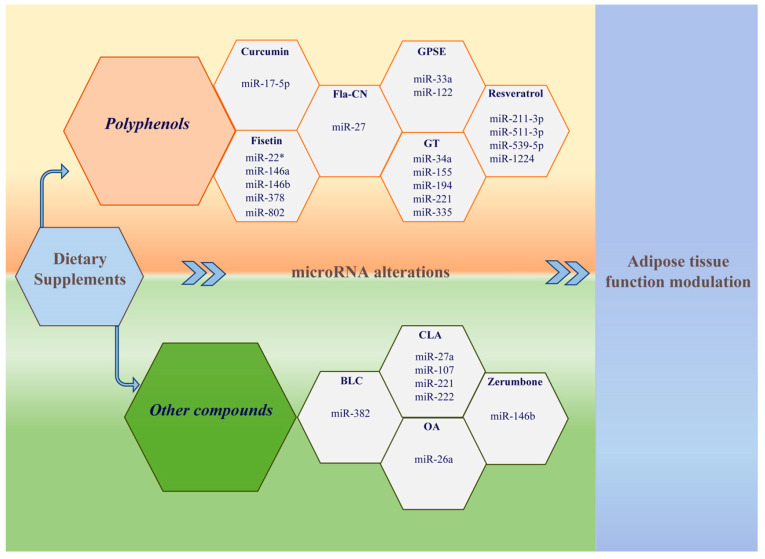
Dietary supplements modify microRNA expression modulating adipose tissue function.

MicroRNAs have emerged as crucial epigenetic modulators in obesity, as well as candidate biomarkers of the metabolic changes occurring after weight loss. Unravelling the complex mechanisms involving microRNAs as regulators of adipose tissue functions is crucial to identify novel therapeutic targets and to develop new strategies for the treatment of obesity. Recently, several DSs, such as polyphenols, FAs, and other plant-derived products, have been demonstrated to modulate adipose tissue metabolism through microRNA expression modifications in animal models of obesity (Figure 1). The evaluated compounds were able to modulate the expression of several microRNAs, which targeted different genes. A possible explanation for these discrepancies lies in the fact that not the same animal models and tissues have been evaluated. However, almost all the emerged microRNA targets are known to play key functions in adipogenesis, lipid metabolism, and glucose metabolism. Overall, they were able to revert obesity-induced microRNA dysregulation, contrasting adipogenesis and leading to weight loss and visceral fat mass reduction. Furthermore, through microRNA expression modulation, they attenuated the most relevant obesity-related metabolic alterations, such as insulin resistance and lipid alterations, in animal models of obesity. Some of them were also able to reduce proinflammatory cytokines in adipose tissue. Interestingly, a wide variety of dysregulated microRNA target pathways, some of them critically involved in adipogenesis, adipocyte differentiation, energy and glucose homeostasis, FA metabolism, and inflammation, have also been identified, significantly contributing to the understanding of the pathophysiological mechanisms underlying obesity and metabolic diseases. A growing number of studies have observed conceivable anti-obesity effects of other substances, such as chitosan, caffeine, capsaicin, and many other plant-derived substances. Future research should evaluate the effect of the administration of these compounds on circulating and tissue microRNA expression as well.

There is consistent evidence in the literature that microRNA expression changes are viable biomarkers for the effect of different intervention strategies in obesity (dietary approaches, bariatric surgery interventions). As for DSs, further evidence from human studies is needed to confirm their possible role as biomarkers of the strength of the anti-obesity effect. 

Indeed, although promising, it should be highlighted that these findings are still not supported by clinical evidence. Even though dietary supplements are not drugs, and generally have a low toxicity profile, there are insufficient data on their possible adverse effects, due to the lack of evidence from clinical trials. Provided that efficacy and safety issues are further evaluated and demonstrated in large human studies, these compounds might represent an attractive adjuvant to conventional therapies for obesity and metabolic disease management. Accordingly, changes in the microRNA profile and target gene expression after the administration of DSs are an interesting field of research and should be evaluated in clinical studies as well, in order to identify novel biomarkers of weight loss and metabolic changes. However, in this perspective, a crucial advance in analytical methodologies for microRNA expression analysis should be achieved, standardizing automatic and reproducible methods for RNA extraction and trying to obtain absolute microRNA quantification to further acquire useful and accurate biomarkers to be used in clinical practice.

## Figures and Tables

**Table 1:** Studies that evaluated microRNA expression changes after administration of polyphenols.

Study	Model	Compound	Tissue/Cell Line	Regulated miRNAs	Target Gene/Pathway
Qin et al. [43]	Male C57BL/6J mouse	Fla-CN	Liver and adipose tissue	miR-27 (↑)	PPAR-γ, C/EBP α, SREBP-1c, FAS (↓ in adipose tissue)
Jeon et al. [44]	Male C57BL/6J mouse	Fisetin	Liver	miR-22*, miR-146a, miR-146b, miR-802, miR-378 (↓)	↓ PGC-1β (miR-378)
Baselga-Escudero et al. [45]	Female Wistar rat	GPSE	Liver	miR-33a, miR-122 (↓)	Lipid metabolism
Gracia et al. [46]	Wistar rat	Resveratrol	VAT	miR-211-3p, miR-1224, miR-539-5p (↑); miR-511-3p (↓)	PPAR-γ, HSL, SP1
Tian et al. [47]	Male C57BL/6J mouse	Curcumin	Epididymal adipose tissue	miR-17-5p (↑)	Adipogenic differentiation
Otton et al. [48]	C57BL/6 mouse	Green tea	Epididymal adipose tissue	miR-155, miR-221, miR-335 (↓)	Adipogenesis
Torres et al. [49]	C57BL/6 mouse	Green tea	Liver	miR-34a (↓), miR-194 (↑)	SIRT1, PPAR-α, Insig2 (miR-34a) Apoa5, Hmgcs2 (miR-194)

HFD: high fat diet; Fla-CN: flavonoid 3-O-[(E)-4-(4-cyanophenyl)-2-oxobut-3-en-1-yl] kaempferol; PPAR-γ: peroxisome proliferator activated receptor-γ; C/EBP α: CCAAT-enhancer-binding protein α; SREBP-1c: sterol regulatory element-binding protein-1c; FAS: fatty acid synthase; PGC-1β: proliferator-activated receptor-γ coactivator-1 β; GPSE: grape seed proanthocyanidin extract; VAT: visceral adipose tissue; HSL: hormone sensitive lipase; SP1: SP1 transcription factor; SIRT1: Sirtuin 1; PPAR-α: peroxisome proliferator activated receptor-α; INSIG2: insulin induced gene 2; APOA5: apolipoprotein A-5; HMGCS2: 3-hydroxy-3-methylglutaryl-CoA-synthase 2.

**Table 2:** Studies that evaluated microRNA expression changes after administration of fatty acids and different plant-derived compounds.

Study	Model	Compound	Tissue/Cell Line	Regulated miRNAs	Target Gene/Pathway
Nazari et al. [50]	Wistar rats	CLA	Adipose tissue	miR-27a (↑)	Adipogenesis
Parra et al. [51]	C57BL/6J mouse	CLA	Retroperitoneal adipose tissue	miR-107 (↓), miR-221 (↑), miR-222 (↑)	Adipocyte differentiation
Chen et al. [52]	C57BL/6 mouse	OA	Liver and Kupffer cells	miR-26a (↑)	Lipid and glucose metabolism
Ahn et al. [53]	C57BL/6N mouse	Zerumbone	Adipose tissue and differentiated 3T3-L1 cells	miR-146b (↓)	SIRT1 (↑)
Choi et al. [54]	Male C57BL/6 mouse	BLC	SAT	miR-382 (↓)	Dio2 (↑)/Adipose tissue browning

CLA: conjugated linoleic acid; OA: oleanolic acid; SIRT1: Sirtuin 1; BLC: ß-Lapachone; SAT: subcutaneous adipose tissue; Dio2: Iodothyronine Deiodinase 2.

## Software

BioRender has been used to generate graphical abstract.
